# Drug Discovery via Human-Derived Stem Cell Organoids

**DOI:** 10.3389/fphar.2016.00334

**Published:** 2016-09-22

**Authors:** Fangkun Liu, Jing Huang, Bo Ning, Zhixiong Liu, Shen Chen, Wei Zhao

**Affiliations:** ^1^Department of Neurosurgery, Xiangya Hospital, Central South UniversityChangsha, China; ^2^Center for Inflammation and Epigenetics, Houston Methodist Research Institute, HoustonTX, USA; ^3^Department of Psychiatry, The Second Xiangya Hospital, Central South University, ChangshaHunan, China; ^4^Mental Health Institute of the Second Xiangya Hospital, Central South University, ChangshaHunan, China; ^5^Chinese National Clinical Research Center on Mental Disorders, ChangshaHunan, China; ^6^Chinese National Technology Institute on Mental Disorders, ChangshaHunan, China; ^7^Hunan Key Laboratory of Psychiatry and Mental Health, ChangshaHunan, China; ^8^Key Laboratory for Stem Cells and Tissue Engineering, Ministry of Education, Sun Yat-sen UniversityGuangzhou, China; ^9^Department of Histology and Embryology, Zhongshan School of Medicine, Sun Yat-sen UniversityGuangzhou, China

**Keywords:** organoid, pluripotent stem cells, intestinal cancer, inflammatory bowel disease

## Abstract

Patient-derived cell lines and animal models have proven invaluable for the understanding of human intestinal diseases and for drug development although both inherently comprise disadvantages and caveats. Many genetically determined intestinal diseases occur in specific tissue microenvironments that are not adequately modeled by monolayer cell culture. Likewise, animal models incompletely recapitulate the complex pathologies of intestinal diseases of humans and fall short in predicting the effects of candidate drugs. Patient-derived stem cell organoids are new and effective models for the development of novel targeted therapies. With the use of intestinal organoids from patients with inherited diseases, the potency and toxicity of drug candidates can be evaluated better. Moreover, owing to the novel clustered regularly interspaced short palindromic repeats/CRISPR-associated protein-9 genome-editing technologies, researchers can use organoids to precisely modulate human genetic status and identify pathogenesis-related genes of intestinal diseases. Therefore, here we discuss how patient-derived organoids should be grown and how advanced genome-editing tools may be applied to research on modeling of cancer and infectious diseases. We also highlight practical applications of organoids ranging from basic studies to drug screening and precision medicine.

## Introduction

The intestinal epithelium is a tissue with an extreme self-renewal capacity fueled by Lgr5^+^ intestinal stem cells (ISCs; Sato et al., 2009; Rookmaaker et al., 2015; Sato and Clevers, 2015; Vetizou et al., 2015). These cells give rise to daughter or progenitor cells that can differentiate into mature epithelial cells required for normal gut function (Sato et al., 2009; Petersen et al., 2014; Sterneckert et al., 2014). Homeostasis of the normal intestinal epithelium is ensured by continuous and rapid turnover of differentiated cells compensated by replication of ISCs which is commonly marked by Lgr5, a seven-transmembrane receptor as a marker of Wnt-regulated adult stem cell populations in the intestine, stomach, pancreas, and prostate (Sato et al., 2009; Snippert et al., 2010; Bas and Augenlicht, 2014; Peterson and Artis, 2014; Vereecke et al., 2014; Kabouridis et al., 2015). Nonetheless, in various pathological conditions, this renewal process can become substantially disordered, resulting in a loss of epithelial integrity, in local inflammation, or even carcinogenesis (Peterson and Artis, 2014; McFarlane et al., 2015; van de Wetering et al., 2015; Amieva and Peek, 2016). Diseases of the intestinal epithelium include chronic disorders such as inflammatory bowel disease (IBD) and gastrointestinal (GI) cancers (Jostins et al., 2012; Mokry et al., 2014; Neurath, 2014; Okamoto and Watanabe, 2015; Stedman et al., 2015). Unfortunately, due to unavailability of effective drugs for treatment of these diseases, the mortality rates remain unacceptably high (Okamoto and Watanabe, 2015). The exceptional costs and the relative paucity of new drug approvals are in large part due to the failure to establish biomimetic platforms in initial *in vitro* screens in order to predict which drugs will perform as intended *in vivo* (Mokry et al., 2014; van de Wetering et al., 2015). Two-dimensional (2D) monocultures of cell lines lose cell-matrix interactions that are necessary to maintain *in situ* phenotypes and thus fail to sustain cellular functions that exist in tissues (Jabaji et al., 2013; Gould et al., 2015). Although animal models recapitulate *in vivo* physiology closely, the most obvious problem is the fundamental difference between animal and human biology (Huch et al., 2013a,b; Karthaus et al., 2014; Nanduri et al., 2014; Gould et al., 2015).

The widespread implementation of organoid technologies provides a more physiologically relevant platform for high-throughput screening during drug discovery (Eglen and Randle, 2015; Walsh et al., 2016). An organoid means *ex vivo* three-dimensional (3D) tissue originating from organ stem cells, embryonic stem cells (ESCs), or induced pluripotent stem cells (iPSCs), with structure and function similar to those of the original organ to some degree (McCracken et al., 2011; Fuller et al., 2012; Lancaster and Knoblich, 2014; Ordonez-Moran et al., 2015; Tamminen et al., 2015; Wieck et al., 2015). So far, various organoid systems have been successfully established from a specific organ and could be expanded infinitely (Sato et al., 2011; Hisha et al., 2013; Mahe et al., 2013; Takebe et al., 2013; Gehart and Clevers, 2015; Rookmaaker et al., 2015; Xinaris et al., 2015; Yin et al., 2016). For intestinal epithelial organoids, also termed the *mini-gut*, tissue stem cells or ESCs/iPSCs can form a crypt-villus structure that mimics *in vivo* gastric, intestinal, or colonic epithelial structures (Stange et al., 2013; DeWard et al., 2014; Watson et al., 2014; Rookmaaker et al., 2015; Salahudeen and Kuo, 2015).

*In vitro* organoids have various advantages over traditional animal models and cell culture systems in human physiological research and disease modeling because (i) an organoid in 3D culture can develop and expand in all directions, thus simulating organ development and morphological features *in vivo* (Mahe et al., 2013; Lancaster and Knoblich, 2014); (ii) an organoid derived from a human organ can maintain its *in vivo* characteristics stably and purely after passaging for many generations without significant genetic or physiological changes (DiMarco et al., 2014; Grabinger et al., 2014); (iii) GI organoid models can be easily established by isolating epithelial crypts from the mouse GI tract or a human GI biopsy, and crypts can grow into crypt-villus structures in less than 7 days; (iv) the clustered regularly interspaced short palindromic repeats/CRISPR-associated protein-9 (CRISPR/Cas9) system has made it feasible to correct or change the human genome *in vitro*, and the off-target effects manifest themselves less during correction of organoid genes (Gao et al., 2014; Gregorieff et al., 2015). For the reasons given above, a 3D organoid has a great potential for drug screening and personalized medicine (Miyoshi et al., 2012; Li et al., 2014; Drost et al., 2015; Janeckova et al., 2015; Kitamura et al., 2015; Oshima et al., 2015; Riemer et al., 2015; van de Wetering et al., 2015). Moreover, organoid buds originating from iPSCs and successful use of CRISPR/Cas9-mediated correction in patients with cystic fibrosis (CF) show great promise for organ transplantation and gene therapy (Eglen and Randle, 2015; van de Wetering et al., 2015; Walsh et al., 2016).

In this review, we discuss the recent developments in gut organoids from primary tissues and pluripotent stem cells. We critically appraise the advantages of organoids as model systems for research into human intestinal diseases related to immunological disorders (Mahe et al., 2013; Peterson and Artis, 2014; Nguyen-Ngoc et al., 2015; Rogoz et al., 2015). We also highlight the potential applications of organoids to clinical drug screening and stem cell transplantation as well as their significance for drug discovery and precision medicine (Mahe et al., 2015; Okamoto and Watanabe, 2015; Schumacher et al., 2015).

## Technologies of Current Intestinal Epithelial Organoid

The most widely used intestinal cellular model is the Caco-2 cell line in 2D culture. An improvement of this model is achieved via cell growth on a microporous membrane allowing for free access of nutrients to either the basolateral or the apical side of the cellular monolayer (Eglen and Randle, 2015; Gould et al., 2015). Another key improved method is co-culture of different cell types in two filters, where they share soluble factors in the medium without a direct physical contact. Nevertheless, intestinal cells growing as a 2D monolayer lack organ-specific microarchitecture and the physiological extracellular matrix microenvironment. Indeed, in drug development, only 5% of the compounds that are found effective in such 2D models reach clinical trials (Eglen and Randle, 2015).

It is likely that 3D culture may provide more reliable cellular models and help to reduce the number of animals used for drug toxicity and efficacy tests (Dekkers et al., 2013; Eglen and Randle, 2015; van de Wetering et al., 2015). Currently, there are several 3D culture methods, including the scaffold-based models (hydrogels or solid biomaterials) and scaffold-free platforms for spheroid growth. In 2009, the first culture system for an intestinal epithelial organoid (mini-gut) was established by means of mouse Lgr5^+^ ISCs (Sato et al., 2009). A single ISC may form 3D crypt structures in a serum-free and mesenchyme-free growth environment containing Matrigel, epidermal growth factor (EGF), Noggin, and R-spondin 1 (Barker et al., 2010; Snippert et al., 2010; Yui et al., 2012; Koo and Clevers, 2014). These mini-guts comprise distinct cell types including Lgr5^+^ ISCs, enterocytes, enteroendocrine cells, goblet cells, and Paneth cells normally present in the gut (Yin et al., 2014). Later, human mini-guts are seeded in a 3D laminin-rich matrix (Mohamed et al., 2014; Jung et al., 2015). Mini-organoids that effectively mimic GI epithelial structure and functional features can be readily generated from crypt stem cells by chelation with 2 mM ethylenediaminetetraacetic acid (EDTA) (Sato et al., 2011). Intestinal crypts are harvested from the human GI tract, then an organoid is grown in the presence of R-spondin, EGF, Wnt3a, Noggin, and other essential growth factors (Peignon et al., 2011; Sato et al., 2011; Demitrack et al., 2015). The crypts form round clear spheres soon after plating, then start to bud continuously 3–5 days later on (Sato et al., 2011). The cell types such as goblet cells, endocrine cells, and enteroendocrine cells appear and surround the central lumen in a single-layer manner, while ISCs and Paneth cells form the bud part of the mini-gut (Jung et al., 2011; Grun et al., 2015; Horvay et al., 2015). In contrast to the structure in the intestine, the ISCs and Paneth cells that are present in the crypt domain and other cells form a villus toward the intestinal lumen (Jung et al., 2011). Usually, organoids are passaged for 7–10 days post-cultivation (Wang et al., 2013). Intestinal organoids can be stably passaged and retain their morphological and genetic characteristics for months and years. Similar protocols and cultivation protocols can be applied to gastric and colon organoids, except that they need additional growth factors like gastrin and FGF10 (Barker et al., 2010; Schumacher et al., 2015; Wroblewski et al., 2015).

Although a mini-gut recapitulates many characteristics of the intestine *in vivo*, the limitations still exist. (i) Mini-guts do not undergo physical stretching that is caused by peristaltic contractions *in vivo* (Barker et al., 2010); (ii) bone morphogenetic protein (BMP) inhibitor Noggin diffuses throughout the organoid from the culture medium; this situation leads to the lack of a BMP signaling gradient in intestinal organoids (Shroyer and Wong, 2007); (iii) in human colon and intestinal organoid culture, Wnt and other factors (such as p38 inhibitor, transforming growth factor beta (TGF-β) inhibitor, and nicotinamide) prevent stem cell differentiation and reduce cell diversity (Miyoshi et al., 2012; de Lau et al., 2014; Germann et al., 2014; Krausova and Korinek, 2014); (iv) intestinal epithelial organoids consist mainly of epithelial cells without interaction with mesenchymal cells (Wilson et al., 2015). This problem can be overcome with embryonic or iPSCs that can form organoids including subepithelial myofibroblasts, immune cells, and enteric nerves (Spence et al., 2011; Takebe et al., 2013).

**FIGURE 1:**
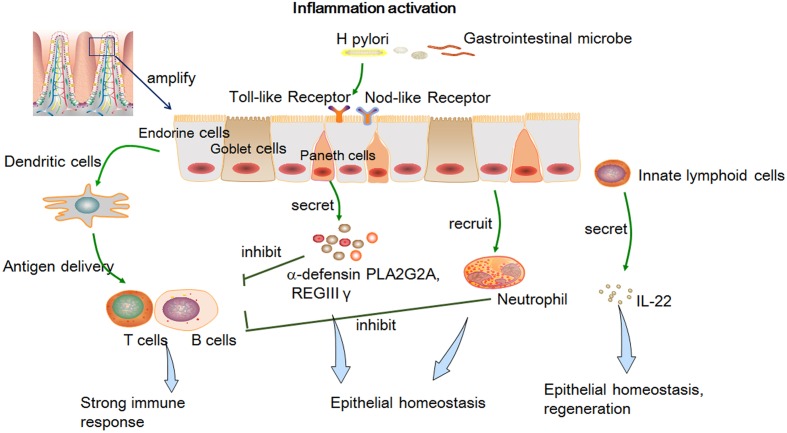
**The epithelial innate immune response and its effect on epithelial homeostasis, regeneration, inflammation, and gastric carcinogenesis after activation of inflammation by a pathogen.** Epithelial cells can express pathogen recognition receptors to pass microbial signals (Nigro et al., 2014; Amieva and Peek, 2016) and generate various peptides against infection and prevent a strong T-cell-mediated immune response (Farin et al., 2014; Leslie et al., 2015); IEC-produced cytokines and dendritic cells can promote T-cell maturation and differentiation (Liu Y. et al., 2015); innate lymphoid cells can produce IL-22 and then regulate ISC survival and proliferation (Bermudez-Brito et al., 2013; Lindemans et al., 2015).

In addition, modeling of intestinal diseases can be improved by introducing additional cell types from lineages other than the epithelium (Lindemans et al., 2015; Vetizou et al., 2015; Pastula et al., 2016). For instance, co-culturing of an intestinal organoid with Paneth cells significantly increases the plating efficiency (Shamir and Ewald, 2014). A microvascular niche has also been successfully generated through co-culturing of tumor cells with stromal cells and endothelial cells (Shamir and Ewald, 2014). To study the complex interaction of intestinal epithelial cells (IECs) and immune cells or microbiota, a model has been optimized to have features similar to those of the *in vivo* system (**Table 1**). An organoid is cultured together with cytokines produced by immune cells or directly with CD8^+^ T cells (Lindemans et al., 2015; Vetizou et al., 2015). This system allows researchers to examine the proliferation, activation, and movement of an epithelial lymphoid *in vitro* (Vetizou et al., 2015). Moreover, enteric pathogens can colonize the GI organoid through microinjection (Bartfeld and Clevers, 2015). The infected models can be genetically manipulated via short hairpin RNA or CRISPR interference single guide RNA afterward, enabling investigators to thoroughly analyze the effects on epithelial homeostasis, regeneration, inflammation, and gastric carcinogenesis (**Figure 1**; Andersson-Rolf et al., 2014; Van Lidth de Jeude et al., 2015).

**Table 1:** Optimization of organoids for immunological research.

*In vitro* models	Research area	Finding	References
Co-culture with lymphocytes	Cancer immunotherapy	Cytotoxic T-lymphocyte-associated protein 4 (CTLA-4) blocker relies on the microbiota in anticancer immunotherapy	Vetizou et al., 2015
Co-culture with interleukin 22 (IL-22) produced by ILCs	Innate immunity and epithelial homeostasis	ILCs induce ISC regeneration and intestinal regeneration though signal transducer and activator of transcription 3 (STAT3) signaling	Lindemans et al., 2015
Gastroid microinjection	Gastroid microbiota	GI microbiota and its effects on epithelial homeostasis, regeneration, inflammation, and gastric carcinogenesis	Hynds and Giangreco, 2013; Foulke-Abel et al., 2014; Nigro et al., 2014; Pham et al., 2014; Zhang et al., 2014; Bartfeld et al., 2015
Stem cell transplantation	Chronic inflammatory disease	Donor stem cells cure DSS colitis and remain functional for over half a year in an animal model	Yui et al., 2012; Fordham et al., 2013


The proof-of-concept use of genome editing in an intestinal organoid was recently demonstrated (Cao et al., 2015; Guye et al., 2016). The mutation (deletion of phenylalanine at position 508) of the CF transmembrane conductance regulator (CFTR) is the primary cause of CF (Liu et al., 2012; Dekkers et al., 2013, 2015; Schneeberger et al., 2015; Than et al., 2016). Intestinal organoids from two CF patients were genome-edited to correct the mutation through CRISPR/Cas9-mediated homology-directed repair (Dekkers et al., 2013; Wiegerinck et al., 2014). Matano et al. (2015) introduced multiple mutations of niche signaling pathways into intestinal epithelial organoids by CRISPR gene editing. Thus, their roles in tumorigenesis and metastases could be elucidated (Matano et al., 2015; Hong et al., 2016; Sakaguchi et al., 2016). Evolutional genome-editing approaches create next-generation organoid systems, advancing the research on intestinal homeostasis, pathogenesis, organoid-based therapies, intestinal neoplasms, and organoid-based drug screening (Fujii et al., 2015; Matano et al., 2015).

## Intestinal Organoids For Research Into Immune Homeostasis Mechanisms

The intestinal immune system must ensure homeostasis by offering adequate protection from pathogens and by remaining tolerant to harmless commensals (McDonald et al., 2008; Neurath, 2014; Peterson and Artis, 2014). Imbalances of immune responses may lead to chronic inflammatory disorders such as IBD (Neurath, 2014; Gao et al., 2015; Amieva and Peek, 2016). The molecular mechanisms of intestinal homeostasis are not fully understood. The model of intestinal organoids co-cultured with immune cells or microorganisms should facilitate identification of molecular mechanisms underlying intestinal homeostasis and interactions of the intestinal epithelial barrier with its environment (Peterson and Artis, 2014; Takahashi et al., 2014; Nozaki et al., 2016).The essential players in intestinal homeostasis are the IECs, derived from Paneth cells, goblet cells, endocrine cells, and enteroendocrine cells (Fuller et al., 2012; Chang et al., 2015; Takebe et al., 2015). They play a crucial role in the innate immune system by directly segregating the internal environment from bacteria and by integrating microbial signals with an innate immune response (Bartfeld et al., 2015; Liu J. et al., 2015). The immune homeostasis effects comprise (i) the epithelial cells that can express Toll-like receptors and other pathogen recognition receptors that can pass a microbial signal on to dendritic cells (Ayabe et al., 2000; Nigro et al., 2014; Amieva and Peek, 2016); (ii) Paneth cells and ISCs can generate various peptides like α-defensin phospholipase A2 group IIA (PLA2G2A) and regenerating islet-derived protein 3 gamma (REGIII-γ) against an infection, and thus prevent a strong T-cell-mediated immune response (Ayabe et al., 2000; Farin et al., 2014; Leslie et al., 2015); (iii) interleukin 8 (IL-8) and tumor necrosis factor alpha (TNF-α) are secreted by IECs when a pathogenic microbe is present and promote dendritic-cell activation (Jostins et al., 2012; Kabouridis et al., 2015); (iv) IEC-produced cytokines and dendritic cells can promote T-cell maturation and differentiation (Liu Y. et al., 2015); (v) innate lymphoid cells (ILCs) can produce IL-22 (when IECs suffer from injury) and then regulate ISC survival and proliferation (Bermudez-Brito et al., 2013; Lindemans et al., 2015). Intraepithelial lymphocytes (IELs) are believed to be necessary for the maintenance and regulation of IECs (Peterson and Artis, 2014). Nonetheless, the molecular mechanisms involved in the interactions between IELs and IECs remain unclear. Dysregulation of this system may contribute to the development of intestinal inflammatory diseases such as celiac disease, IBD, and intestinal cancer (Neurath, 2014; Sugimoto et al., 2015).

To effectively study the correlation between immune cells and IECs, several modified organoid systems have been built that directly apply immune cells or a cytokine generated by immune cells to research under pathological or physiological conditions (Neurath, 2014). For example, Nozaki et al. (2016) showed that IELs can be maintained with epithelial organoids in an IL-2-, IL-7-, and IL-15-supplemented medium. Fluorescent imaging revealed an active, multidirectional movement of IELs along the surface of IECs and their migration relative to organoid structures (Nozaki et al., 2016). Using a GI organoid and an IL-22 co-culture system, Lindemans et al. (2015) found that immune molecules can directly promote the growth of ISCs to enhance epithelial regeneration. In the co-culture system, IL-22 secreted by ILCs can induce intestinal organoid growth after injury in the intestine. IL-22 targets signal transducer and activator of transcription 3 (STAT3) signaling and leads to organoid formation and ISC regeneration *in vivo*. This mechanism was also proven to be independent of Paneth cells, which means that ILCs themselves play an important part in the intestinal barrier repair after damage. This study provides solid evidence that the immune system can maintain and promote intestinal regeneration (Gilbert et al., 2015; Lindemans et al., 2015; Zachos et al., 2015).

The intestinal organoids could be infected with intracellular pathogens, and this approach may help to identify the molecular mechanisms governing microbiota–epithelial interactions (Foulke-Abel et al., 2014; Pham et al., 2014). For example, Finkbeiner et al. (2012) reported application of the intestinal organoid system to a model of rotavirus infection. In addition, a homologous human rhinovirus is more infectious than a heterologous one (Saxena et al., 2015). Recently, Wilson et al. (2015) used an organoid system to explore the mechanisms behind *Salmonella enterica* infection. They showed that organoids form a sealed lumen that contains high concentrations of α-defensins capable of restricting the growth of *S. enterica* for at least 20 h (Wilson et al., 2015). Similarly, Forbester et al. (2015) showed that *Salmonella* Typhimurium microinjected into the lumen of intestinal organoids can invade the epithelial barrier. On the other hand, a mutation causing a defect in the *Salmonella* pathogenicity island 1 invasion apparatus yields a pathogen less capable of invading the organoid epithelium (Forbester et al., 2015).

It was also shown that inside the intestinal organoid, bacteria in the gland induce a much stronger T-cell response and inflammation than bacteria in the surface layer (Foulke-Abel et al., 2014; Bartfeld et al., 2015; Zachos et al., 2015). On the one hand, the mucus layer may protect epithelial cells from bacteria (Okamoto and Watanabe, 2015). On the other hand, the host restricts receptors initiating the nuclear factor kappa-light-chain-enhancer of activated B cells (NF-κB) activation in the deeper glands, which are less in contact with bacteria. A study by Bartfeld et al. (2015) supports the notion that the epithelial barrier can separate the bacteria that colonize mucosal surfaces from iron during the inflammation process; this mechanism represents ancient innate immune defense against infection. In the gastric mucosa, transferrin, hemoglobin, ferritin, and neutrophils secreting lactoferrin can bind free iron. In addition, inflammation upregulates hepcidin, which reduces iron uptake in the small intestine (Bartfeld et al., 2015). Antimicrobial proteins secreted by Paneth cells can also prevent a strong T-cell response on mucosal surfaces (Keshav, 2006).

Although the microbiota poses a threat to an intestinal organoid, the host–pathogen interactions also have a beneficial effect on gut epithelial homeostasis (Foulke-Abel et al., 2014). Bacterial products may interact with the host and potentially modulate an innate immune response and epithelial regeneration (Foulke-Abel et al., 2014). Nigro et al. (2014) found that within an intestinal organoid, Lgr5^+^ stem cells express the cytosolic innate immune sensor Nod2 more than it is expressed in Paneth cells, and microbiota-derived molecules can trigger Nod2 secretion, which is beneficial for ISC proliferation.

Besides physiological relevance, the intestinal organoid microbiota model has several other advantages. CRISPR/Cas9-mediated genome editing in intestinal organoids enables the studies on gene function under intestinal homeostatic and pathophysiological conditions (Schwank et al., 2013; Matano et al., 2015). Furthermore, this system may serve as a promising tool in studies on individual host–microbiota interactions using human biopsy samples (Nigro et al., 2014; Zhao et al., 2015).

## The Use of Intestinal Organoids For Therapeutic Purposes

Intestinal organoids have been transplanted into a damaged colon for tissue repair (Yui et al., 2012; Fordham et al., 2013; Spurrier and Grikscheit, 2013; Chen Y. et al., 2015; Elliott et al., 2015; Joly et al., 2015). Fukuda et al. (2014) described methods for long-term expansion of Lgr5^+^ cells in culture. They reintroduced Lgr5^+^ cell-derived colon organoids into a superficially damaged mouse colon, and found that the organoid cells formed a single-layered functionally and histologically normal epithelium (see also Barthel et al., 2012; Yui et al., 2012; Gehart and Clevers, 2015). Watson et al. (2014) showed that an *in vivo* transplant of ESC- or iPSC-derived human intestinal organoids results in marked expansion and maturation of the epithelium and mesenchyme.

**FIGURE 2:**
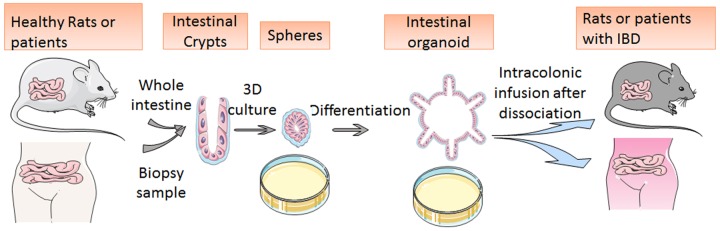
**Schematic diagram of mini-gut culture and application to stem cell transplantation.** The image shows organoid development from isolated crypts to round spheres and budding organoids. After the cultured and dissociated organoids are transplanted into animal models or human bodies, they spontaneously move toward the damaged focus of the DSS-colitis ulcer and promote reconstruction of the epithelial structure.

In the treatment of IBD, traditional anti-TNF-α therapy based on the anti-immunologic theory has shown limited long-term therapeutic responses; accordingly, a stem cell transplantation therapy involving a 3D-cultured organoid may solve the problem (Yui et al., 2012; Spurrier and Grikscheit, 2013; Fukuda et al., 2014; Mokry et al., 2014; Okamoto and Watanabe, 2015). Cultured organoids have been proven to regenerate damaged epithelial cells *in vivo* after trans-anal transplantation (Fordham et al., 2013). Researchers upregulated ILCs in the GI organoid and then dissociated them into small pieces for infusion. After the cultured and dissociated organoids are transplanted into an animal model of dextran sulfate sodium (DSS)-colitis, they spontaneously move toward the damaged focus of the DSS-colitis ulcer and promote reconstruction of the epithelial structure (Fordham et al., 2013). The newly produced epithelial cells retain their normal functions of the barrier against infection and of nutrition absorption; these findings mean that the DSS-caused colitis may be cured (Fordham et al., 2013). The transplanted cells stay functional for more than half a year. Consequently, these results indicate that ISC transplantation may also be adapted to patients with IBD and elicit a long-term response (**Figure 2**; Yui et al., 2012; Fordham et al., 2013). Further studies also revealed that intestinal organoids can regenerate the colonic mucosa after inflammation.

## Engineered Cancer Organoids

Human colorectal carcinoma (CRC), a common cancer type, evolves from adenomatous polyps to advanced adenomas, carcinomas *in situ*, and adenocarcinomas (Drost et al., 2015). Mutations in APC, AXIN2, or CTNNB1 are believed to change a proliferating tissue into invasive and ever-expanding neoplasm that shows submucosal invasion and causes systemic metastases (Matano et al., 2015). In the research on human colon cancer, conventional cancer cell lines and animal models have long been used for cancer modeling, tumorigenesis studies, and drug screening. Cell lines cannot form the authentic tissue structure and acquire mutations after passaging, whereas animal models take a long time and do not fully reflect genetic characteristics of human tissues (van de Wetering et al., 2015). Organoids either from human biopsy samples or from iPSCs may be a solution to these problems (van de Wetering et al., 2015).

van de Wetering et al. (2015) established a living cancer biobank of colon organoids derived from either cancer tissue or adjacent healthy tissue from 20 patients with CRC. Whole-exome sequencing has been performed on these models along with tissues from biopsies. They found that the subtypes can be identified by means of an organoid model of an individual patient and that *in vitro* models can fully recapitulate the original genetic and morphological characteristics (van de Wetering et al., 2015). In addition, porcupine inhibitor was tested and found to be effective against an organoid with special alteration of RNF43 (van de Wetering et al., 2015). Furthermore, 35 medications used for the treatment of CRC have been tested in the biobank, and their specific targets and possible resistance were thoroughly analyzed. This effort may point to an effective strategy for a precision therapy via drug screening and gene analysis and correction (van de Wetering et al., 2015).

Additionally, genetic engineering by means of the CRISPR/Cas9 system has been applied to the remodeling of colorectal cancer (Onuma et al., 2013; Li et al., 2014; Drost et al., 2015; Matano et al., 2015; Salahudeen and Kuo, 2015). When APC, TP53, KRAS, and SMAD4 genes are knocked out in a cultured human colon organoid, the engineered organoid can grow independently from niche factors and shows tumor morphology and invasive features after a transplant into an animal (Drost et al., 2015). Moreover, after these driver genes are altered in an adenoma organoid originating from patients, the latter dramatically propagates and yields distant metastases (McCracken et al., 2014). According to the results of Drost et al. (2015), chromosomal instability and aneuploidy can be caused and mainly mediated by APC and TP53, which are important for malignant transformation into CRC. In addition, when an APC mutation is corrected in a colon adenoma or adenocarcinoma, the epithelial differentiation is rescued and preserved, and the homeostasis is then restored (Dow et al., 2015).

Nevertheless, the cancer that is engineered in an organoid possesses aberrations only in driver genes, not in DNA methylation, and mainly progresses along the adenoma route (Dow et al., 2015). When transplanted into a renal subcapsular region in animals, the engineered organoid does not expand or metastasize effectively as compared with the original-CRC-derived organoid (Salahudeen and Kuo, 2015). In agreement with the findings of Drost et al. (2015), an organoid line with a quadruple knockout of APC, TP53, KRAS, and SMAD4 shows only limited capacity for invasion into the cancer stroma. To solve these problems, additional modifications such as epigenetic changes and chromosomal instability should be introduced into the existing engineered models. Possibly, a newly designed model containing all these oncogenic factors and mutations will acquire both histological features and powerful proliferative and metastatic abilities.

## Drug Screening

Newly developed medical treatments of human diseases usually have limitations such as individual differences among patients, difficulty with prediction of outcomes, and time-consuming drug testing (Eglen and Randle, 2015; Martin, 2015; Sampaziotis et al., 2015; Dedhia et al., 2016). Precision medicine is now coming into focus and becoming more relevant to clinical practice (Mou et al., 2015; VanDussen et al., 2015). 3D organoid culture based on a specific disease and even on a specific individual is expected to develop into a powerful tool of precision therapy (Hynds and Giangreco, 2013; Eglen and Randle, 2015; Walsh et al., 2016). Primary cancers, infectious diseases, and developmental diseases can be replicated *ex vivo* on biopsy samples from patients, as is the case for the biobank of colon cancer organoids (van de Wetering et al., 2015). These kinds of “live” clinical specimens may become useful for drug testing, gene editing, or for research on prognosis.

Prostate cancer organoids, generated from castration-resistant Nkx3.1-expressing cells, have also been tested in modeling of a drug response (Chua et al., 2014). A tumor organoid originating from a mouse or human tissue or after oncogenic transformation can maintain genetic and phenotypic characteristics of its progenitor; accordingly, the Akt inhibitor MK-2206 and the mechanistic target of rapamycin (mTOR) inhibitor ridaforolimus have been proven to be significantly effective against organoid formation; these findings are consistent with the already-known AR and PI(3)K signaling activation in prostate cancer (Chua et al., 2014). Another group studied advanced prostate cancer models based on castration-resistant prostate cancer and found that a patient-derived organoid can recapitulate the *in vitro* results, and genetic manipulations hold promise (Gao et al., 2014). All these results suggest that an *ex vivo* prostate organoid can– potentially be used for drug research.

Organoids from primary human pancreatic ductal adenocarcinoma (PDAC) have been used to identify new effective drugs (Huang et al., 2015; Nielsen et al., 2016). Patient-derived tumor organoids show similar histoarchitecture features and differentiation markers expression level with primary tumor (Huang et al., 2015). Even histological features like different populations of invasive glands could be conserved in tumor organoids (Huang et al., 2015). Five human tumors with a poor response to chemotherapy were selected, and the organoid models that were derived from them were tested with epigenetically targeted drugs. As a result, some of the organoids showed decreased proliferation after treatment, thus indicating new therapeutic targets in this cancer. Because PDAC patients harbor numerous genetic and epigenetic changes, organoid models may be a good solution for fast drug selection and validation.

As for other solid tumors such as glioblastoma, primary cancer cell lines can be isolated from patients, and then they could be made into patient-derived xenograft models or directly cultured in a 3D matrix (Eglen and Randle, 2015). Unlike slices of tumor samples, the tumor cell lines may provide more dynamic and detailed clinical information and could be applied to analysis of cancer-associated signaling pathways (Shamir and Ewald, 2014). When digested and cultured in a 3D matrix, a tumor organoid from different sources shows varied morphological features and indolent or high invasion, in accordance with a clinical prognostic outcome. After treatment with radiation, different glioblastoma lines showed varied chemotherapeutic drug responses, with implications for clinical practice (Shamir and Ewald, 2014).

For other 3D tumor organoids such as breast cancer organoid and lung cancer organoid, although no clinical trials are underway before now, drug screening platforms through organoid proved to be a useful preclinical model for pharmacodynamic profiling of human tumors (Vaira et al., 2010; Rookmaaker et al., 2015; Walsh et al., 2016). All these approaches to analysis of (and intervention into) diseases by means of an organoid system should expand the basic knowledge about the relevant diseases and improve the management of patients.

## A Summary and Future Directions

*In vitro* organoid models, with the capacity for rapid growth, stable differentiation, and suitability for non-mutational and genetic manipulations, have become an effective tool for human disease research and a powerful weapon of precision therapy (de Lau et al., 2012; Spurrier and Grikscheit, 2013; Forster et al., 2014; Todhunter et al., 2015). The culture system can consist of a mixture of already existing internal cells such as endothelial cells, Paneth cells, or peripheral T cells, or alternatively, growth factors secreted by internal functioning cells in a complete culture medium. Eventually, the organoid is expected to acquire characteristics similar to those of a healthy or diseased organ (Moore et al., 2014; Sugimoto et al., 2015; Overeem et al., 2016). The CRISPR/Cas9 system also enables researchers to implement gene modifications *in vitro*, which are hard to accomplish directly in a human body (Guye et al., 2016). Therefore, wild-type or disease-causing genes along with their downstream effects can be analyzed in a more rapid and reliable manner, and the off-target effects manifest themselves less after correction of genes in an organoid (Lutsenko, 2016). Genetically modified organoids have been successfully transplanted into a damaged colon for tissue repair and function recovery. Besides, research on engineered cancer together with clinical genome databases can be harnessed to deliver disease-causing genes into a mini-organ and induce it to proliferate into invasive and metastatic cancer organoid lines that even cause similar systemic metastasis after they are returned into the human body (Kazanjian and Shroyer, 2011; Cancer Genome Atlas Network, 2012; Cooks et al., 2013). A large database of human individualized diseases has been created and studied via generation of tissue organoids based on biopsies. All these methods contribute to research on tumorigenesis and drug screening (de Lau et al., 2012; Basak et al., 2014; DeWard et al., 2014).

Surely, the organoid system has its limitations. First, the limited number of cell types in intestinal epithelial organoids and the absence of the immune, nervous, and vascular system in cultured intestinal organoids result in drug effects that are different from those *in vivo* (Fuller et al., 2012; Jabaji et al., 2013; Grun et al., 2015). Additionally, although the main cell types of epithelial cells that are generated in organoids are similarly diverse in comparison with those found *in vivo*, the 3D organization cannot be spatially and structurally similar to an *in vivo* murine intestine or consistent with other organoids (Onuma et al., 2013; Wieck et al., 2015). Third, the microenvironment and hormone levels, pH, and the health status of the human body as well as epigenetic factors cannot be fully reproduced in an *ex vivo* organoid (Lancaster and Knoblich, 2014; Dekkers et al., 2015; Wroblewski et al., 2015). In addition, a wider range of disease databases needs to be established and extensively tested (Stedman et al., 2015; Xinaris et al., 2015; Fatehullah et al., 2016).

To solve these problems, a new disease profile of an organoid model and an effective co-culture system that mimics the *in vivo* intestinal system are necessary (Lancaster and Knoblich, 2014; Chen E.C. et al., 2015; Rookmaaker et al., 2015; Salahudeen and Kuo, 2015; Tamminen et al., 2015). Recently, a variety of intestinal culture systems combined with nerve or mesenchymal cells have been established (Takebe et al., 2015; Pastula et al., 2016). Furthermore, additional functional models involving an organoid and a pathogen should be devised to identify various pathways leading to human infectious diseases (Yin et al., 2016). Especially for chronic inflammation, new models are necessary to obtain more direct and convincing evidence for more detailed research into the role of chronic inflammation in carcinogenesis (Watson et al., 2014; Wiener et al., 2014; Sinagoga and Wells, 2015). Infectious organoids can also be genetically manipulated to study genes and epigenetic factors in the development of inflammation (Fujii et al., 2015; Lutsenko, 2016). The CRISPR/Cas9-mediated correction has been successfully applied to the CFTR mutation for the treatment of patients with CF (Durand et al., 2012; Liu et al., 2012; Schwank et al., 2013; Martin, 2015; Mou et al., 2015; Dedhia et al., 2016). Identification and correction of candidate genes for targeted therapy may open up new opportunities for treatment of immune diseases. Biobanks of either oncological or inflammatory diseases pave the way for new drugs to clinical practice (Eglen and Randle, 2015; Martin, 2015; Mou et al., 2015; Walsh et al., 2016).

Researchers also need to analyze signaling pathways leading to NF-κB activation in systems that inhibit bacterial proliferation (Wroblewski et al., 2015; Guye et al., 2016). Regarding the ISC transplantation therapy, further studies are needed to identify a possible pathogen and to clarify the effects on organoids and on the intestinal mucosa (Forster et al., 2014; Todhunter et al., 2015; Xinaris et al., 2015). After a transplant into mice, intestinal organoids can acquire villus-like structures that may be modified for future clinical applications. For precision therapy and drug screening, the organoid models for testing need to be created and standardized more quickly to better uncover gene–drug associations and to obtain more accurate prognostic information. The new choice of a 3D culturing matrix and detailed protein composition may reflect natural organ microenvironments better. Finally, as data on organoid responses during drug screening and data on genetic profiles are generated, it is necessary to analyze their positive correlations for timely clinical decisions and for prediction of responses to treatment.

## Author Contributions

FL and JH reviewed literature and prepared the manuscript. BN drew the figures and revised the manuscript. ZL revised the manuscript. SC and WZ supervised all the work.

## Conflict of Interest Statement

The authors declare that the research was conducted in the absence of any commercial or financial relationships that could be construed as a potential conflict of interest.
